# Evaluation of emotion processing in HIV-infected patients and correlation with cognitive performance

**DOI:** 10.1186/2050-7283-1-3

**Published:** 2013-02-27

**Authors:** Eleonora Baldonero, Nicoletta Ciccarelli, Massimiliano Fabbiani, Manuela Colafigli, Erika Improta, Alessandro D’Avino, Annalisa Mondi, Roberto Cauda, Simona Di Giambenedetto, Maria Caterina Silveri

**Affiliations:** 1 Institute of Clinical and Infectious Diseases, Catholic University of the Sacred Heart, Rome, Italy; 2 Memory Clinic, Catholic University of the Sacred Heart, Rome, Italy

**Keywords:** Emotions, HIV, Neurocogntive correlates of emotions, HIV-related factors and emotions

## Abstract

**Background:**

Facial emotion recognition depends on cortical and subcortical networks. HIV infection of the central nervous system can damage these networks, leading to impaired facial emotion recognition.

**Methods:**

We performed a cross-sectional single cohort study consecutively enrolling HIV + subjects during routine outpatient visits. Age, gender and education-matched HIV-negative healthy individuals were also selected. Subjects were submitted to a Facial Emotion Recognition Test, which assesses the ability to recognize six basic emotions (disgust, anger, fear, happiness, surprise, sadness). The score for each emotion and a global score (obtained by summing scores for each emotion) were analyzed. General cognitive status of patients was also assessed.

**Results:**

A total of 49 HIV + and 20 HIV − subjects were enrolled. On the Facial Emotion Recognition Test, ANOVA revealed a significantly lower performance of HIV + subjects than healthy controls in recognizing fear. Moreover, fear facial emotion recognition was directly correlated with Immediate Recall of Rey Words. The lower the patients’ neurocognitive performance the less accurate they were in recognizing happiness. AIDS-defining events were negatively related to the correct recognition of happiness.

**Conclusions:**

Fear recognition deficit in HIV + patients might be related to the impaired function of neural networks in the frontostriatal system. AIDS events, including non-neurological ones, may have a negative effect on this system. Inclusion of an emotion recognition test in the neuropsychological test battery could help clinicians during the long term management of HIV-infected patients, to better understand the cognitive mechanisms involved in the reduction of emotion recognition ability and the impact of this impairment on daily life.

## Background

Facial emotion recognition depends on a large number of different cortical and subcortical structures which participate in recognizing emotions shown on the face. When an emotionally meaningful stimulus is presented, information is first scanned along the occipital and temporal neocortex, where perceptual information is extracted from the face. Then, after ≈ 100 ms, the stimulus is categorized as expressing an emotion or not, based on the structural properties of the image. The amygdala and orbitofrontal cortices might participate in the facial emotion recognition process in at least three different ways. First, they might modulate perceptual representations via feedback. This mechanism could contribute to fine-tuning the categorization of the facial expression and allocating attention to some of its features. Second, the amygdala and the orbitofrontal cortices might trigger associated knowledge, projecting to other regions of the neocortex and the hippocampal formation. This mechanism could contribute specifically to the retrieval of conceptual knowledge about the emotion. Third, these structures might generate an emotional response in the subject through connections to motor structures, the hypothalamus and brainstem nuclei, where the components of an emotional response to facial expression can be activated. This mechanism could contribute to generating knowledge about another person’s emotional state via the process of simulation, drawing on the somatosensory-related cortices in the right hemisphere to represent the emotional changes in the perceiver (see [1] for a review).

Several studies have demonstrated disrupted facial emotion recognition abilities in patients with Parkinson’s disease [2–4], Huntington’s disease [5, 6], and obsessive compulsive disorder [7], consistently with dysfunction of the frontostriatal pathway and amygdala [8, 9].

We wanted to analyze whether facial emotion recognition is also impaired in HIV + patients, as this pathology primarily involves the frontostriatal connections [10] and the temporal limbic structures [11].

The severity of HIV-associated neurocognitive disorders has been significantly reduced thanks to combination antiretroviral therapy (cART) [12–14], even if milder forms of neurocognitive disorders still persist. This can be ascribed to a possible neurotoxicity of antiretrovirals on cognitive functions [15], to cardiovascular risk factors [16, 17], or to the natural effect of aging [18].

Notably, some features of the neuropsychological impairment observed in HIV-infected populations have been associated with HIV-related frontostriatal abnormalities [10], suggesting that these difficulties are caused by the neuropathological process of HIV infection of the central nervous system (CNS) [19]. As mentioned above, these neural structures, which are involved in the recognition of basic facial emotions, interact within a larger cortico-limbic system [1]. Although atrophy of the temporal and limbic lobes has also been described in HIV-infected patients [20] this finding has not been confirmed in other study [21].

If impairment of the frontostriatal connections and temporal limbic structures is a typical expression of HIV pathology, we would expect that HIV infected patients, compared to an healthy population of subjects, performed worse on emotion recognition tasks, as described in a recent paper [22]. Moreover, we hypothesize that the severity of the HIV pathology (quantified by HIV-related variables such as CD4 cell counts or past AIDS-defining events) could be related to the severity of the emotion recognition impairment, consistently with previous evidence [23].

## Methods

### Subjects

We performed a cross-sectional single-cohort study. HIV-infected neurologically asymptomatic patients were consecutively enrolled during routine outpatient visits from April 2010 to May 2011. Exclusion criteria were age below 18 years, active or known past CNS opportunistic infections, history of neurological disorders, active psychiatric disorders, alcoholism or drug abuse, and linguistic difficulties for non-native patients.

Demographic, clinical and laboratory variables were collected for each subject at the time of the neuropsychological examination.

CNS penetration-effectiveness (CPE) rank was calculated for each antiretroviral regimen according to the CHARTER group criteria revised in 2010 [24].

We also selected an age, gender and education-matched HIV-negative healthy control population (HC), which included 20 subjects. HC had no history or risk factor for neurological impairment and were not taking any medication deemed to affect cognitive abilities. They had no history for HCV infection and were not past injecting drug users. Moreover, they had no clinical or anamnestic evidences of depression. They were recruited among students above 18 years of age, hospital personnel, or patients’ caregivers or relatives. All subjects were volunteers. They received no financial remuneration for participating.

### Standard protocol approvals, registrations, and patient consents

The research design and protocol received ethical approval from University of Sacred Heart-Rome Ethics Committee. Informed consent was obtained from all participants according to the Helsinki Declaration.

### Neuropsychological examination

All patients were administered a comprehensive neuropsychological battery to assess general cognitive status. The following areas were investigated: memory by means of the Immediate and Delayed recall of Rey’s words [25], Digit and Spatial Span [26]; attention and executive abilities by means of the Stroop test [27], Trail-making test B [28], Drawings [29], and Multiple Features Target Cancellation (MFTC) [30]; language by means of the Phonological Fluency test [25]; and speed of mental processing by means of the Wechsler Adult Intelligence Scale (WAIS) digit symbol [31] and the Grooved Pegboard Test [32]. Patients’ scores on each test of the neuropsychological battery were adjusted for age, gender and education based on normative data available for the Italian population. Patients were diagnosed with Asymptomatic Neurocognitive Impairment (ANI) if they scored 1SD below the normative cut-off in two or more domains according to standard criteria [33]. To obtain an evaluation of global cognitive performance, the total number of pathological tests was calculated for each patient.

The Zung Self-Rating Depression Scale [34] and the Instrumental Activities of Daily Living (IADL) scale [35] were also administered.

We assessed patients’ and controls’ facial emotion recognition using Ekman and Friesen’s series [36]. On the Facial Emotion Recognition Test, photographs of the faces of ten people (six female, four male) corresponding to each of six basic emotions (disgust, anger, fear, happiness, surprise, sadness) were given. Participants were asked to select the emotion represented on the face from six emotion labels displayed below each face. Responses were given orally and recorded by the examiner. Participants could view the facial stimuli until they gave the response with no time limitations.

A score of 1 was assigned for each correct response. The scores for each emotion category and a global score (obtained by summing scores for each emotion) were calculated.

### Data analysis

Inspection of emotion global score distribution with the Kolmogorov–Smirnov Test of Normality revealed that data were normally distributed (p = 0.34). Therefore, statistical analyses were conducted using parametric tests on the raw emotion scores.

Performance of patients and controls on the Facial Emotion Recognition Test was assessed by carrying out a mixed design ANOVA with group (HIV, HC) and emotion (disgust, anger, fear, happiness, surprise, sadness) as categorical factors, and each emotion score as dependent variable. According to the recommendations for exploratory analyses [37], effect sizes were computed in addition to p values to determine meaningful effects for the emotion processing data.

The correlation between facial emotion recognition accuracy and cognitive performance was assessed using the Pearson product–moment correlation coefficient. To control for the probability of committing a type I error in multiple comparisons, the Bonferroni correction was adopted by setting the p value at ≤0.004.

For HIV-infected patients, standard linear regression analyses were used to determine the extent to which HIV-related variables or the total number of pathological neuropsychological tests affected emotion recognition accuracy for each emotion and the emotion global score. Variables showing a p value < 0.1 associated with the outcome in the univariate analysis were then investigated in a multivariate model.

Several studies showed that age [38] and depression levels [39] have an impact on emotion recognition abilities; according to these studies, we included this demographic and clinical variables as covariates in multivariate analysis.

All analyses were performed using the SPSS version 13.0 software package (SPSS Inc., Chicago, IL).

## Results

### Characteristics of patients and controls

A total of 49 HIV-infected patients were enrolled; their main demographic and clinical characteristics are reported in Table 1. At the time of the neuropsychological examination, 46 (93.9%) participants were on cART. IADL score was at ceiling for all patients (8/8).

**Table 1:** **Personal and clinical data of patients (n = 49) and controls (n = 20)**

	Patients	Controls	***p***
	No. (%) or median (IQR)*	No. (%) or median (IQR)*	
Male	40 (81.6)	16 (80)	0.87
Age, y*	49 (43–55)	48.5 (44–54)	0.88
Education, y*	13 (8.5–17)	13 (11.3–14.6)	0.85
Pathological Zung depression scale	5 (10.3)		
Transmission risk factor:			
Heterosexual	15 (30.6)		
Injecting drug users	7 (14.3)		
Homosexual	20 (40.8)		
Unknown	7 (14.3)		
Time from HIV diagnosis, y*	14 (3.5–18.1)		
HCV co-infection	9 (18.4)		
Past AIDS-defining events	9 (18.4)		
Past suboptimal therapy	19 (38.8)		
Off Therapy	3 (6.1)		
Time from starting last cART regimen, y*	1.6 (1.1 – 3.1)		
Time from starting first cART regimen, y*	10.2 (3.1 – 14.6)		
CPE rank*	6 (4–7)		
HIV-RNA < 50 copies/mL	44 (89.8)		
CD4 cell count, cells/μL*	570 (437–734)		
CD4 cell count nadir, cells/μL*	211 (115–314)		

Abbreviations: No: number; IQR: interquartile range; y: years; cART: combined antiretroviral therapy; CPE: penetration effectiveness score; HCV: hepatitis C virus.

Overall, 28.6% of the patients showed an ANI, as assessed by the neuropsychological battery; the others 65.3% showed no cognitive impairment; 3 patients (6.1%) did not perform the neuropsychological battery. On the Zung Self-Rating Depression Scale, 10.2% of the patients obtained a pathological score.

Patients and controls were matched for age, gender and education (p value of t-test and chi square reported in Table 1).

### Performance on the facial emotion recognition test

Analyses of HIV and HC groups’ performances on the Facial Emotion Recognition Test revealed a significant main effect of group [F (1, 402) = 6.44, p = 0.011], a significant main effect of emotion [F (5, 402) = 21.35, p < 0.001] and a significant group by emotion interaction [F (5, 402) = 6.40, p < 0.001]. To examine the interaction effect, we conducted a post hoc Fischer LSD test, which showed that HIV-infected patients were significantly less accurate than HC in identifying fearful expressions (p < 0.001). Moreover, HIV infected patients [β = −2.37, 95% confidence interval (CI) -3.42 to −1.32, p < 0.001] confirmed to have a worse performance on fear recognition after adjusting for age and education in a multivariate linear regression model.

**Figure 1:**
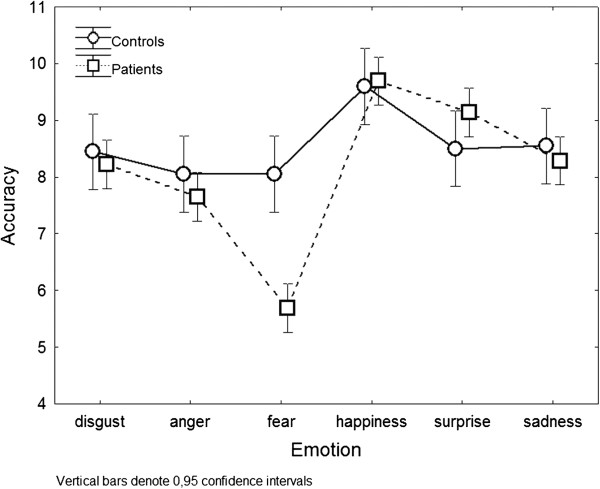
**HIV and HC groups’ accuracy on each emotion of the facial emotion recognition test.**

The groups did not significantly differ in their ability to recognize any other emotion (Figure 1).

Mean raw scores of the HIV and HC groups and the Effect Size values for each emotion category are shown in Table 2.

**Table 2:** **Performance of HIV (n = 49) and HC (n = 20) groups on the facial emotion recognition test**

	HIV	HC	***p***	EFFECT SIZE
	Mean (sd)	Mean (sd)		Partial Eta squares values
Emotion Global Score	50.0 (3.8)	48.7 (5.2)	0.32	0.010
Disgust score	8.2 (1.5)	8.4 (1.1)	0.57	0.005
Anger score	7.6 (1.8)	8.0 (1.1)	0.32	0.012
Fear score	5.6 (2.3)	8.0 (1.3)	< 0.001	0.208
Happiness score	9.7 (0.9)	9.6 (0.6)	0.81	0.003
Surprise score	9.1 (1.3)	8.5 (1.1)	0.11	0.052
Sadness score	8.2 (1.5)	8.5 (1.4)	0.51	0.006

To determine whether the fear recognition deficit was an expression of the cognitive deficit, we divided the HIV + group into two subgroups: ANI group and patients who showed no cognitive impairment. T-test for independent samples showed that both subgroups performed significantly worse than the HC group in fear recognition [ANI vs. HC: 5.38 (2.3) vs. 8.05 (1.35), p = 0.001; not cognitively impaired HIV + vs. HC: 5.9 (2.42) vs. 8.05 (1.35), p < 0.001].

### Correlation between fear facial emotion recognition score and performance on neuropsychological tests

For HIV-infected patients, we conducted a correlation analysis between their Fear Facial Emotion Recognition score and scores obtained on each test of the neuropsychological battery (see Table 3).

**Table 3:** **Correlation among the Fear Facial Emotion Recognition and the Neuropsychological tasks in the HIV group**

	Fear score
**Memory**:	
Immediate recall of Rey’s words	0.42*
Delayed recall of Rey’s words	0.34
Digit span (forward)	0.05
Spatial span (forward)	0.12
**Attention and executive abilities**:	
Stroop test (errors)	0.03
Stroop test (time)	0.04
Trail-making test B (errors)	−0.11
Trail-making test B (time)	−0.18
Drawings	−0.06
MFTC	0.11
**Language**:	
Phonological fluency	0.10
**Speed of mental processing**:	
WAIS digit symbol	0.18
Grooved Pegboard (dominant hand)	−0.22
Grooved Pegboard (nondominant hand)	−0.13

Values represent Pearson’s coefficients.

*significant correlation for p < 0.004 (two-tailed) (Bonferroni correction).

Fear facial emotion recognition was directly correlated with Immediate Recall of Rey Words.

### Relationship between emotion recognition and HIV-related factors

The potential influence of HIV-related factors on emotion recognition abilities was investigated by linear regression analysis: in particular, we explored the relationship between HIV-related variables, the Facial Emotion Recognition global score and each emotion category score. The number of pathological scores on the neuropsychological examination was considered an index of HIV-related neurocognitive impairment. In the multivariate analysis, only lower education level was independently associated with a worse facial emotion recognition global score (β = 0.53, 95% CI 0.15 to 0.91; p = 0.007) after adjusting for age, total number of pathological scores and CD4 cell counts at nadir. Patients with past AIDS-defining events (β = −0.75, 95% CI-1.45 to −0.06; p = 0.035) and a higher total number of pathological scores on the neuropsychological test battery (β = −0.24, 95% CI −0.41 to −0.06, p = 0.008) showed poorer ability to recognize happiness, when scores were adjusted for age and depression levels.

No other variable was associated with any other emotion category when adjusted for age and depression levels.

## Discussion

Similar to previous studies [22, 23], we found that HIV-infected patients performed worse than HC in recognizing the facial emotion of fear. This deficit does not seem related to severity of the cognitive impairment; in fact, patients with ANI performed as accurately as patients who had no documented cognitive deficit.

It has been suggested that specific deficits in the recognition of different categories of facial emotions may reflect task difficulty factors [40]. This is also supported by cross-cultural studies in healthy subjects showing that accuracy in recognizing happiness is high (94% correct responses on emotion recognition tasks) and fear is low (70%) [41, 42]. On the other hand, there are considerable evidence that the correct recognition of facial expression of fear depends on specific neural structures, that seems to have a critical role in mediating the autonomic and behavioural responses associated with this emotion [1, 43].

In agreement with the results of cross-cultural studies [41, 42], in our study both patients and controls performed worse when they had to recognize fear than the other emotions, confirming that this emotion might be more difficult to recognize than the others. On the other hand, fear was also the only emotion on which patients performed significantly worse than controls. This would suggest that impairment of neural substrates that are supposed to be specifically involved in HIV pathology, could concur in fear recognition deficit in addition to the effects of task difficulty. Previous studies [23] suggest in fact, that fear recognition abnormalities in HIV may be due to a disruption of the broader neural network involved in emotion recognition which depends on the integrity of frontal system. Unfortunately, we do not have direct evidence of the involvement of such substrates in our population. However, since such an impairment is currently demonstrated [44] we cannot exclude that the low performance in fear recognition obtained by our patients could be due at least in part to the effect of infection.

If this were true, demonstration of the presence of impaired fear recognition should be considered an early marker of cognitive impairment in HIV population. Studying fear recognition in patients with other brain pathologies that spare the frontal regions as well as neuroimaging studies [11, 44] on HIV + patients could contribute to clarify this issue.

Multivariate analysis demonstrated an independent relationship between severity of cognitive impairment and score on recognizing the emotion of happiness, that is, patients with a higher number of pathological scores on the neuropsychological examination were less accurate in recognizing happiness. Moreover we observed an association between the global emotion recognition score and education level, finding that did not emerge in previous studies [45]. These results confirm that recognizing emotions requires the integrity of “high level” cognitive abilities, as already reported in patients with neurodegenerative diseases (see [46] for a review).

The emotion happiness was also found in association with HIV-related variables. In particular, happiness was independently and inversely associated with past AIDS-defining events.

The association between recognition of happiness and general neurocognitive impairment as well as the association between recognition of happiness and past AIDS-defining events, could lead us to hypothesize that a deficit in recognizing this emotion might emerge only in subjects in more severe stages of HIV pathology in agreement with a recent study [22] demonstrating that the ability to discriminate between levels of happiness intensity on facial expression was specifically altered in HIV patients with impaired neurocognitive performance.

We acknowledge that our study might have some limitations because uncontrolled biases can occur in cross-sectional surveys performed in routine clinical practice. Furthermore, we cannot exclude a possible confounding effect of different IQ on emotion recognition, although there is no reason to assume a different distribution of IQ value in patients and controls. Moreover, although not significant, the performance of HC group in recognition of facial expression of surprise was worse than HIV patients; since the effect size for this emotion was moderate, we cannot exclude potential power issues. Anyway, both groups obtained high scores, so data observed might be attributed to a randomness. At last, we did not consider either the psychological and social implications of HIV infection and their possible impact on emotion recognition ability [47] or the psychological premorbid characteristics.

## Conclusion

In conclusion, a deficit in facial emotion recognition was confirmed in HIV-infected patients. For some emotions, a relationship has been demonstrated with variables related to severity of the HIV infection and global cognitive performance, whereas for some other variables (in particular fear) a potential contribution of an asymptomatic cerebral involvement cannot be excluded. Inclusion of an emotion recognition test in the neuropsychological test battery could help clinicians in the long term management of HIV-infected patients, to better understand the cognitive mechanisms involved in the reduction of emotion recognition ability and the impact of this impairment on daily life.

## Funding

This research was supported by an unrestricted grant from Abbott Virology.

## Abbreviations

cART: Combination antiretroviral therapy
CNS: central nervous system
CPE: CNS penetration-effectiveness
HC: HIV-negative healthy control population
MFTC: Multiple Features Target Cancellation
WAIS: Wechsler Adult Intelligence Scale
ANI: Asymptomatic Neurocognitive Impairment
IADL: Instrumental Activities of Daily Living
CI: Confidence interval.
